# Hints to the H.I.N.T.S. Exam for Acute Vestibular Syndrome

**DOI:** 10.1212/NE9.0000000000200248

**Published:** 2025-09-10

**Authors:** Harry W. Sutherland, Christine E. Gummerson

**Affiliations:** From the Department of Neurology, Yale School of Medicine, New Haven, CT.

Sudden-onset, constant vertigo with nausea/vomiting, gait unsteadiness, and spontaneous nystagmus define acute vestibular syndrome (AVS). This Figure details the Head-Impulse-Nystagmus-Test-of-Skew (HINTS) exam, which uses special maneuvers to identify central etiologies of AVS with greater sensitivity than hyperacute MRI.^1,2^ The presence of unidirectional nystagmus (especially when following Alexander's law) and catch-up saccades following a lateral head impulse (from difficulty maintaining target fixation) both typically imply peripheral pathology.^1^ Vertical ocular misalignment (skew deviation) on cross-cover testing is always suspicious for central pathology.^1^ Central pathology cannot be excluded if any individual finding is inconsistent with peripheral localization.^1^

**Figure:**
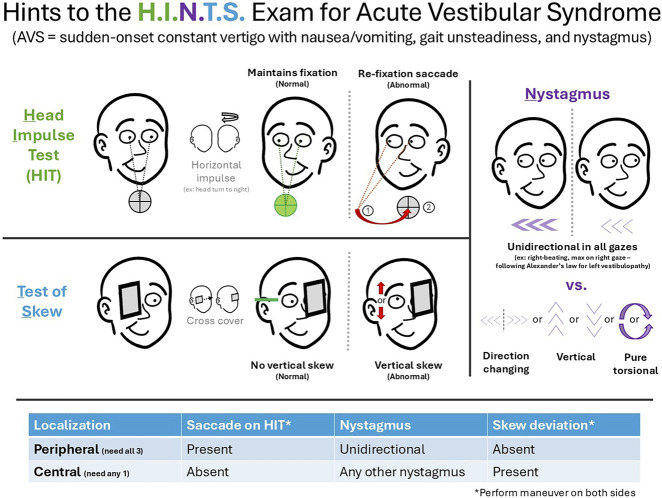
Hints to the H.I.N.T.S. Exam for Acute Vestibular Syndrome
